# Effect of *FCGR* polymorphism on the occurrence of late-onset neutropenia and flare-free survival in rheumatic patients treated with rituximab

**DOI:** 10.1186/s13075-017-1241-0

**Published:** 2017-03-07

**Authors:** Sofia Ajeganova, Daniel Tesfa, Hans Hägglund, Bengt Fadeel, Inger Vedin, Anna Linda Zignego, Jan Palmblad

**Affiliations:** 1 Department of Medicine Huddinge, Karolinska Institutet, 14186 Stockholm, Sweden; 2 Center for Hematology and Regenerative Medicine, Department of Medicine Huddinge, Karolinska Institutet, 14186 Stockholm, Sweden; 3 Medical Affairs, Roche AB, 10074 Stockholm, Sweden; 4 Department of Hematology, Uppsala University Hospital, 75185 Uppsala, Sweden; 5 Unit of Molecular Toxicology, Institute of Environmental Medicine, Karolinska Institutet, 171 77 Stockholm, Sweden; 6 Center for Systemic Manifestations of Hepatitis Viruses, Department of Internal Medicine, University of Florence, 50134 Florence, Italy

**Keywords:** Late-onset neutropenia, Rituximab, *FCGR*, *BAFF*, Polymorphism, Rheumatic disease

## Abstract

**Background:**

The causes and mechanisms of late-onset neutropenia (LON) following rituximab treatment in patients with rheumatic diseases are not known. In this study, we aimed to investigate the role of established Fcγ receptor gene (*FCGR*) polymorphisms and B-cell-activating factor (*BAFF*) gene promoter polymorphisms for the development of LON and for the efficacy of rituximab in patients with rheumatic diseases.

**Methods:**

A single-center case-control retrospective study was nested in a cohort of 214 consecutive patients with rheumatic diseases treated with rituximab. Eleven patients presented with LON. Fifty non-LON control subjects were matched by diagnosis, age, sex, and treatments. Single-nucleotide polymorphisms of *FCGR* (*FCGR2A* 131H/R, *FCGR2B* 232I/T, *FCGR3A* 158V/F) and *BAFF* promoter polymorphism −871C/T were analyzed with polymerase chain reaction-based techniques, and serum immunoglobulin M (IgM) and BAFF levels were analyzed by enzyme-linked immunosorbent assay. Flare-free survival was related to LON occurrence and polymorphisms.

**Results:**

The *FCGR3A* V allele, but not other *FCGR* polymorphisms, correlated with the occurrence of LON; each V allele conferred a fourfold increased OR for LON (*p* = 0.017). *FCGR3A* 158V/V and presentation with LON were associated with a longer flare-free survival (*p* = 0.023 and *p* = 0.031, respectively). *FCGR3A* 158V/V was related to lower IgM levels (*p* = 0.016). Serum BAFF levels showed no relationship with LON and *BAFF* −871C/T promoter polymorphism. There was a tendency toward longer flare-free survival in patients with the *BAFF* −871T/T allotype compared with the C/T or C/C allotypes (*p* = 0.096).

**Conclusions:**

The results of the present study suggest that presentation with LON may be a result of the intrinsic efficacy of rituximab in patients with rheumatic diseases. LON could indicate a longer biological and therapeutic activity of rituximab modulated by a certain genotypic polymorphism: the high-affinity *FCGR3A* V allele. This genotype and the occurrence of LON are both related to longer flare-free survival, suggestive of common mechanisms for LON and duration of response to rituximab. The role of the *BAFF* −871C/T promoter polymorphism in LON occurrence is unclear.

## Background

Late-onset neutropenia (LON) is now recognized as one of the significant side effects of rituximab therapy that may lead to serious infections. LON is defined as an absolute blood neutrophil count <1.5 × 10^9^/L occurring 4 weeks after the last rituximab infusion. The frequency of LON in hematological malignancies and autoimmune diseases is comparable. LON has been reported in 5–27% of patients with lymphoma treated with rituximab [1, 2] and in 1.5–23% of patients with rheumatic diseases [3–6]. Because of the morbidity associated with LON, it is important to understand the causes and mechanisms of this phenomenon.

The binding of rituximab to Fcγ receptors (FcγRs) of macrophages and natural killer (NK) cells is supposed to influence therapeutic efficacy of rituximab [7]. A certain single-nucleotide polymorphism (SNP) in the *FCGR* gene, the *FCGR3A* 158V/V genotype (also called the *176V/V genotype*), enhances ligation of rituximab to this receptor [8, 9], and it has been associated with improved clinical response in non-Hodgkin lymphoma and rheumatoid arthritis (RA) [10–12], as well as with B-lymphocyte depletion in patients with systemic lupus erythematosus (SLE) [13]. The clinical relevance of other *FCGR* genotypes is not known.

After depletion, B lymphocytes return to the peripheral blood after a mean of 8 months [14]; this return is preceded by increases in blood levels of B-cell-activating factor (BAFF, also called *B-lymphocyte stimulator* or *BLyS*). BAFF, a cytokine expressed mainly by neutrophils and monocytes, plays a central role in the stimulation of B-lymphocyte proliferation, differentiation, immunoglobulin production, and survival [15]. Elevated serum BAFF levels have been correlated to a poorer clinical outcome in patients with rheumatic diseases [16, 17]. Thus, regulation of BAFF expression may affect the efficacy of rituximab. Enhanced generation of BAFF has been associated with the presence of a certain SNP of the *BAFF* gene promoter (the −871C/T genotype) [18–20]. Recently, the *BAFF* −871C/T promoter polymorphism has been reported to influence the response to rituximab in patients with RA [20, 21].

Predisposing factors for LON and the potential clinical consequences of LON are poorly defined. We therefore conducted a nested case-control analysis of a cohort of rituximab-treated patients with rheumatic diseases. We hypothesized that three established *FCGR* polymorphisms—*FCGR2A* 131H/R, *FCGR2B* 232I/T, and *FCGR3A* 158V/F—and the *BAFF* −871C/T promoter polymorphism are related to occurrence of LON through modified therapeutic activity of rituximab. We thus investigated the possible predictive role of the polymorphisms for LON. Second, in this study with an extended initial control population [3], we examined the relationship between LON and serum levels of immunoglobulin M (IgM) and also BAFF levels. Finally, we asked whether the efficacy of rituximab differs between patients with LON and those without LON.

## Methods

### Study design and patients

**Fig. 1:**
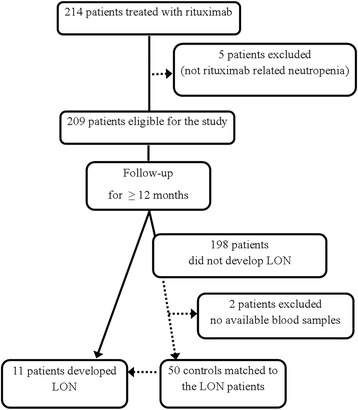
Chart of the selection of the study population. *LON* Late-onset neutropenia

All 214 consecutive adult patients treated with rituximab for rheumatic diseases from June 2003 until March 2009 and followed for at least 12 months at the Department of Rheumatology, Karolinska University Hospital Huddinge, Stockholm, Sweden, qualified for inclusion in the study. The medical records were retrospectively reviewed until December 2011 and also 2 years prior to initiation of rituximab. Five cases in which neutropenia could have developed as a consequence of the autoimmune disorder per se or prior therapy were excluded. (For details, see [3].) Overall, 11 cases of LON were identified at routine follow-up or emergency visits, as previously reported [3]. LON occurred after the first rituximab treatment in seven cases and after the second in four cases. The control subjects (50 non-LON patients) were drawn from the whole study population after the diagnoses and were representative of and proportional to the LON cases (i.e., SLE, antineutrophil cytoplasmic antibody-associated vasculitis [AAV] and RA) with available blood samples. The control patients were matched by age, sex, and previous and concurrent treatments to the patients with LON (Fig. 1 and Table 1). The diagnoses of SLE, AAV, and RA satisfied American College of Rheumatology classification criteria [22–24].

**Table 1:** Characteristics of 61 patients, by group according to occurrence of late-onset neutropenia during follow-up

	Total (*n* = 61)	LON (*n* = 11)	Non-LON (*n* = 50)	*P* value
Age at rituximab start, years, mean (SD), range	57.3 (16.4), 18–85	58.9 (18.2), 29–82	57.0 (16.2), 18–85	0.73
Female sex, %	75.4	72.7	76.0	0.82
Disease characteristics				
Diagnosis				0.82
SLE, %	23.0	27.3	22.0	
AAV, %	21.3	27.3	20.0	
RA-seropositive, %	37.7	36.4	38.0	
RA-seronegative, %	18.0	9.1	20.0	
Disease duration, years, median (IQR)	7.0 (3.0–13.0)	4.0 (2.0–11.0)	7.5 (3.0–13.3)	0.43
Previous treatments				
Number of DMARDs, mean (SD), range	3.6 (1.8), 0–8	4.1 (2.1), 1–8	3.5 (1.8), 0–7	0.27
Cytotoxics and biologics, %	63.9	81.8	60.0	0.30
Prednisolone, %	98.4	100	98.0	1.0
Major indication for rituximab, by manifestations				0.15
Arthritis and dermatologic, %	59.0	45.5	62.0	
Renal, %	19.7	9.1	22.2	
Pulmonary and ENT, %	16.4	27.3	14.0	
Neurologic, %	3.3	9.1	2.0	
Hematologic, %	1.6	9.1	0	
Rituximab regimens				0.84
375 mg/m^2^ weekly for 4 weeks, %	14.8	9.1	16.0	
1 g twice 2 weeks apart, %	59.0	63.6	58.0	
0.5 g twice 2 weeks apart, %	26.2	27.3	26.0	
Concurrent DMARDs				
Any DMARDs, %	78.7	72.7	80.0	0.69
Cyclophosphamide, %	21.3	27.3	20.0	
Azathioprine, %	9.8	9.1	10.0	
Mycophenolate mofetil, %	8.2	9.1	8.0	
Methotrexate, %	34.4	9.1	40.0	
Concurrent prednisolone, %	88.5	100	86.0	0.33

The *p* values refer to the comparisons between LON and non-LON groups. Cytotoxics used were cyclophosphamide and chlorambucil

*Abbreviations: LON* Late-onset neutropenia, *SLE* Systemic lupus erythematosus; *AAV* Antineutrophil cytoplasmic antibody-associated vasculitis, *RA* Rheumatoid arthritis, *DMARDs* Disease-modifying antirheumatic drugs, *ENT* Ear, nose, throat

### Rituximab therapy

Details of the therapies and patient selection are given in Table 1 and Fig. 1. Briefly, all patients received rituximab because of the inefficacy of other immunosuppressives or contraindications for standard therapies. Initiation and dosing of rituximab were based on the discretion of the treating physician. A rituximab infusion of 375 mg/m^2^ weekly for 4 weeks was offered for patients with SLE and patients with AAV, and two doses of 1 g or 0.5 g were given to patients with RA. Steroid and immunosuppressive regimens were sequentially reduced as clinically needed.

Patients were assessed according to a standard care protocol, which included history taking, a physical examination, and laboratory tests at the decision to treat or before first administration of rituximab. Assessments were repeated each 3–6 months during follow-up, with additional visits scheduled as clinically needed. For patients who experienced flares, standard therapy was initiated, such as retreatment with rituximab or other biologics, disease-modifying antirheumatic drugs (DMARDs), and/or glucocorticoids. Retreatment with rituximab because of flare was never initiated before 12 months from the previous rituximab treatment cycle. Blood samples for biobanking were collected at baseline and 3, 6, and 12 months of follow-up.

### Time to flare

Time to flare was defined as the time from the day of rituximab initiation to the time of recurrence or new symptoms that warranted therapy escalation beyond a temporary increase in the glucocorticoid dosage. For the purpose of rituximab response analysis, follow-up after a rituximab cycle leading to neutropenia was censored at the time of flare or retreatment with rituximab or other agents; otherwise, follow-up was ended at the time of the last evaluation in December 2011.

### Laboratory tests

*G*enotyping was performed in all 61 patients. Genomic DNA was isolated from peripheral blood mononuclear cells using QIAamp® DNA Blood Mini Kit according to the recommendations of the manufacturer (Qiagen, Hilden, Germany). *FCGR* genotyping of the SNPs 158V/F (rs396991) in the *FCGR3A* gene and 131H/R (rs1801274) in the *FCGR2A* gene was performed with allelic discrimination using two TaqMan assays (C_9077561_10 and C_25815666_20, respectively; Applied Biosystems, Foster City, CA, USA) with supplied probes and primers on a Rotor-Gene 6000 real-time system (Qiagen). *FCGR2B* 232I/T (rs1050501) genotyping was performed using oligonucleotide probing based on fluorescence resonance energy transfer technology as previously described [25]. *BAFF* −871C/T (rs9514828) genotyping was performed by restriction fragment length polymorphism analysis. The primers used for *BAFF* promoter amplification were 5′-GGCACAGTCAACATGGGAGT-3′ (forward) and 5′-GCTAAGTGTTTTAGCATTGAATTG-3′ (reverse) as previously described [26]. The polymerase chain reaction products were subjected to a restriction enzyme-based screening of −871C/T using 20 U of BsrBI restriction enzyme. All genotyping results were in consistent with Hardy-Weinberg equilibrium, and genotyping efficiency was validated (Haploview v.4.1 software; Broad Institute, Cambridge, MA, USA). All samples were run in duplicates-triplicates.

Serum levels of human BAFF were determined by using the Quantikine BAFF enzyme-linked immunosorbent assay (R&D Systems, Abingdon, UK) according to the recommendations of the manufacturer. Serum immunoglobulin levels were assessed according to standard clinical procedures.

### Statistical methods

The chi-square and two-sided Fisher’s exact tests were used for comparisons as suitable. The Wilcoxon matched pairs signed-rank test was used to analyze within-group changes at follow-up. The relationship between gene polymorphisms and LON occurrence was analyzed by Spearman’s correlation analysis and logistic regression. Analyses of serum IgM levels over time were done with linear mixed models. The Kaplan-Meier method with the log-rank test was applied to determine the difference between flare-free survival 12 months following rituximab in the LON and non-LON groups. The duration of follow-up for the survival analyses was chosen on the basis of mean period of rituximab-induced B-lymphocyte depletion in patients with rheumatic disease [14].

## Results

The studied population comprised 61 patients, 11 of whom experienced LON during follow-up and 50 matched control patients who did not (non-LON) (Table 1). Demographic and disease characteristics did not differ between the groups (Table 1). At the initiation of rituximab therapy, patients were a mean (SD) of 57.3 (16.4) years old and had a median disease duration of 7 years. In addition to rituximab, all patients were given immunotherapy with DMARDs and/or prednisolone. The patients were followed for at least 12 months and up to 5.6 years after rituximab therapy.

### Genotype distributions in patients with LON and control subjects without LON

**Fig. 2:**
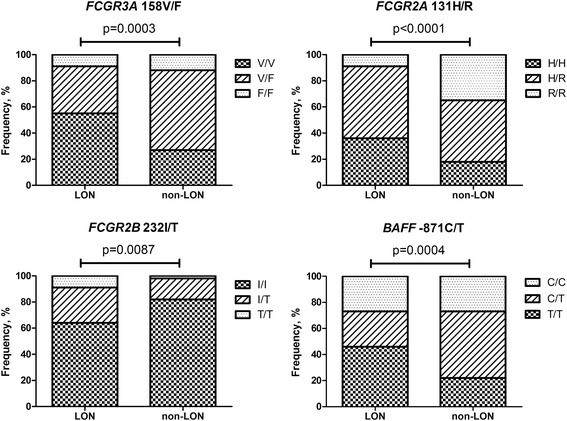
Distribution of *FCGR* and *BAFF* promoter gene polymorphisms in the patients who developed late-onset neutropenia (LON) during follow-up and the matched control patients who did not (non-LON). *BAFF* B-cell-activating factor

The distribution of genotypes differed significantly between LON and non-LON groups (Fig. 2).

Among patients who developed LON, 55% were homozygous for the high-affinity V allele of the *FCGR3A* gene, as compared with 27% of non-LON patients (*p* = 0.086). The number of V alleles was significantly correlated to LON (*r* = 0.42, *p* = 0.01). Next, we examined how much each V allele affected the risk for LON development. We found that each additional V allele was associated with a fourfold increased risk of LON (OR 4.0, 95% CI 1.0–16.7, *p* = 0.017).

Even if the number of cases was limited, no statistically significant associations were found between LON and the *FCGR2A* 131H/R or *FCGR2B* 232I/T genotype. Of note, there was a contrasting relationship in the distribution of the *FCGR2B* and the *FCGR3A* genotypes in the LON and non-LON groups (Fig. 2). Thus, the high-affinity *FCGR3A* 158V/V allotype was more frequent, whereas the low-affinity *FCGR2B* 232I/I allotype was less frequent, in the LON group than in the non-LON group. The same pattern also yielded the distributions of V/F versus I/T and F/F versus T/T between the groups. The *BAFF* −871T/T genotype, which is associated with higher BAFF levels [14, 15], was more frequent in the LON group than in the non-LON group (45% vs. 24%; *p* = 0.12) (Fig. 2).

### Serum BAFF levels and *BAFF* promoter polymorphism

**Fig. 3:**
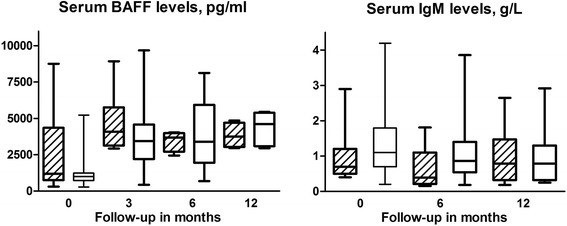
Box plots (median, minimum, and maximum) of serum B-cell-activating factor (BAFF) and immunoglobulin M (IgM) levels after rituximab treatment during follow-up by late-onset neutropenia (LON) occurrence. *Diagonal lined boxes* represent the LON group, and *open boxes* represent the non-LON group. The numbers of patients with available serum for measurement of serum BAFF levels in the LON group were 11 at treatment initiation, 7 at 3 months, and 4 at 6 and 12 months; in the non-LON group, the respective numbers of patients with BAFF measurements were 48, 39, 24, and 5. The numbers of patients with measured IgM serum levels in the LON group were 11 at baseline, 10 at 6 months, and 8 at 12 months; the respective numbers of patients in the non-LON group were 47, 42, and 22

After rituximab treatment, there was an increase of serum BAFF levels at 3 months and a decrease thereafter. There was no statistically significant difference in serum BAFF levels at baseline and after 3 and 6 months, or for changes from baseline to follow-up, between the LON and non-LON groups (Fig. 3). We then analyzed the relationship between the *BAFF* −871T/T genotype and serum BAFF levels. In both the LON and the non-LON groups, we observed a trendwise positive association between possession of the T allele (compared with the C allele) and an increase in serum BAFF levels from 0 to 3 months (*r* = 0.27, *p* = 0.073).

### Serum immunoglobulin levels in relation to LON and *FcγR* and *BAFF* polymorphisms

We previously showed that patients with rheumatic diseases who developed LON after rituximab treatment had lower serum IgM levels for a longer period than matched non-LON control subjects [19]. In the present study, the non-LON control group was extended. Baseline IgM levels were similar in the LON and non-LON groups. However, compared with the non-LON control subjects, patients with LON had a more pronounced decrease in IgM levels from baseline to the lowest values in the first year after rituximab treatment (median (IQR)﻿−0.37 [-0.25 to- 0.60] g/L vs. −0.2 [-0.01 to - 0.50] g/L, *p* = 0.042). The previous observation of lower serum IgM during follow-up in the patients who developed LON was hence confirmed.

Because enhanced binding of rituximab to FcγRs might confer more pronounced and/or prolonged IgM reduction, and because recovery of immunoglobulin production is partly governed by BAFF [13], we next examined whether changes in serum IgM levels were related to *FCGR* and *BAFF* genotypes. There was no significant difference in IgM levels in the *FCGR3A* 158V/V, V/F, and F/F groups at baseline. However, patients homozygous for the high-affinity V allele of *FCGR3A* had lower IgM levels over time than those with the low-affinity F allele, independently of sex and previous treatments (β = −0.30, 95% CI −0.54 to −0.06; *p* = 0.016). We found no significant association between IgM levels and other tested *FCGR* and *BAFF* promoter polymorphisms. No association was found between serum immunoglobulin G (IgG) levels at baseline or over time with LON occurrence or the genetic polymorphisms.

### Time to flare in relation to LON and *FCGR* and *BAFF* polymorphisms

Because of the higher affinity of the Fc portion of anti-CD20 monoclonal antibodies on FcγRs, more efficient binding and activation of NK cells are rationales for both the occurrence of neutropenia in the context of rituximab and the clinical response to rituximab. Therefore, we asked if LON is associated with clinical benefit as a longer time to flare. Because B-lymphocyte depletion and clinical effect following rituximab commonly lasts <12 months [14], evaluation was done at 12-month follow-up visits.

**Fig. 4:**
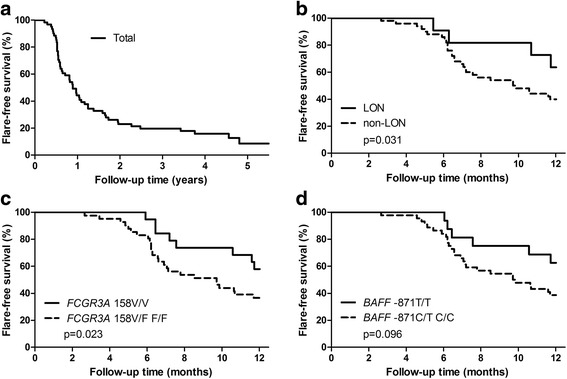
Kaplan-Meier estimates of the percentages of patients without flare from the time of rituximab therapy in total and by groups of late-onset neutropenia (LON) occurrence and genotypic polymorphism. Depicted are flare-free survival estimates for 61 patients after treatment with rituximab through the observation period (53 flares) (**a**) and during the 12-month follow-up (34 flares) in the groups by occurrence of LON (**b**), by *FCGR3A* 158V/F polymorphism (**c**), and by B-cell-activating factor (*BAFF*) −871C/T polymorphism (**d**)

Of all patients, 87% experienced flares during 5 years of follow-up; the proportions were 86% in the LON group and 91% in the non-LON group (Fig. 4a). The median times to flare were 12 (8.0–19.5) months in the LON group and 7.5 (6–13) months in the non-LON control group (*p* = 0.22). There were fewer patients in the LON group who experienced flares within 12 months (36.4%) than in the control group (60.0%) (*p* = 0.15).

At the 12-month assessment, LON occurrence was positively correlated to time to flare (*r* = 0.27, *p* = 0.043). The presence of LON was associated with lower odds of flare (OR 0.10, 95% CI 0.01–0.92; *p* = 0.028). Figure 4b presents the Kaplan-Meier estimates of the percentages of patients with flare-free follow-up in the LON and non-LON groups. Patients who developed neutropenia had a longer flare-free survival than the control subjects without it (*p* = 0.031).

Considering the relationship between the *FCGR3A* 158V/V genotype and the occurrence of LON shown in our analysis, we found, as expected, a significant positive correlation between this allotype (vs. V/F or F/F) and time to flare (*r* = 0.29, *p* = 0.039). Possession of the V allele was negatively associated with flare at 12-month assessment (OR 0.10, 95% CI, 0.03–0.42, *p* = 0.034). The Kaplan-Meier curves illustrate the difference in flare-free survival by possession of the *FCGR3A* 158V/V polymorphism (*p* = 0.023) (Fig. 4c). No association between other tested *FCGR* polymorphisms and flare-free survival was found, whereas patients with the *BAFF* −871T/T allotype tended to have a longer flare-free survival than those with the *BAFF* −871C/T or C/C allotype (*p* = 0.096) (Fig. 4d).

## Discussion

In this prospective case-control analysis nested within a large cohort of rituximab-treated patients with rheumatic diseases, we examined possible genetic bases for the occurrence of LON and the consequences of presentation with that complication in the largest dataset of reported LON cases reported to date. We showed, for the first time to our knowledge, the relationship between the occurrence of LON and possession of the high-affinity *FCGR3A* V allele. We also confirmed a previous observation of lower serum IgM levels in patients with LON than in those who did not develop LON. Further, an association between the *FCGR3A* V allele and lower serum IgM levels was demonstrated. Finally, we observed a favorable association between both LON and the *FCGR3A* V allele and a longer duration of flare-free survival following rituximab therapy. Taken together, these findings suggest that the variation in quality of clinical response to rituximab may be explained by genotypic polymorphism in a certain FcγR, and that a more pronounced B-cell depletion could likely account for both occurrence of LON and longer therapeutic activity of rituximab. Although patients with the *BAFF* −871T/T allotype had a longer flare-free survival than patients with the *BAFF* −871C/T or C/C allotype, no significance of serum BAFF levels related to LON occurrence was found.

LON represents a potentially severe clinical phenomenon occurring after rituximab treatment in patients with lymphoma and patients with rheumatic disease. At the time of LON occurrence, up to a 90% infection rate is reported in patients with rheumatic disease diagnoses, and in many patients granulocyte colony-stimulating factor (G-CSF) is required [3, 11]. It is therefore of clinical importance to define predisposing factors for LON. To some extent, this has been investigated in patients with lymphoma. Thus, high cumulative doses of myelotoxic agents, including autologous stem cell transplant, have been proposed as a predictive factor for LON [27–29].

The biological rationale to investigate *FCGR* polymorphisms is based on the hypothesis that LON is related to higher binding of the Fc part of rituximab to the FcγR on macrophages and NK cells, conferring an increased efficacy of the antibody-dependent cellular cytotoxicity (ADCC) process that leads to depletion of B lymphocytes. The FcγRIIIa and FcγRIIa receptors, which mainly mediate proinflammatory activity, as well as the FcγRIIb receptor with anti-inflammatory activity, may be of significance for the ADCC process [30]. *FCGR3A* V allotype is the most thoroughly investigated and is known to display higher affinity for IgG1 antibody (such as rituximab) than the *FCGR3A* F allotype. Patients carrying the V/V genotype, compared with the V/F and F/F genotypes, demonstrate higher biological and clinical responses to rituximab in the treatment of B-lymphoproliferative malignancies, RA and SLE [9, 13, 31].

In addition to SNPs in the *FCGR* gene, the role of copy number variation has been proposed to be related to the efficacy of antibody-based drugs [32] and differential susceptibility to autoimmune diseases [33]. *FCGR* gene copy number variation was not assessed in our sample. Investigation of whether the *FCGR* gene copy number genotypic profile is associated with occurrence of LON requires larger study populations.

To the best of our knowledge, this report is the first regarding the relationship between LON occurrence and possession of the high-affinity *FCGR3A* V allele in patients with rheumatic disease. This finding is in line with a report on patients with lymphoma in which the *FCGR3A* 158V/F polymorphism was suggested as a novel risk factor for LON [34, 35]. We also extended our analysis to several FcγR types. In this context, the role of possession of the anti-inflammatory FcγRIIb was of particular interest, not only because of its largely opposing effects compared with FcγRIIIa in mediating actions of antibodies but also because it is expressed mainly on B lymphocytes [30, 36]. The significance of polymorphisms in the *FCGR2A* and *FCGR2B* genes for LON occurrence was not documented here, however. We acknowledge that our analysis had limited statistical power to estimate the contribution of the *FCGR2B* 232I/I polymorphism; taking into account its prevalence in the LON and non-LON groups, a sample size of 96 patients in each group would have been required to detect a difference with 80% statistical power (based on the normal approximation to the binomial distribution). Considering the low numbers of patients in our study, the negative findings do not exclude a role of other polymorphisms and FcγRs for developing LON. Of note, we observed differences in the distribution of *FCGR* polymorphisms in the LON and non-LON groups, with more frequent *FCGR2A* 131H/H and *FCGR2B* 232I/I polymorphisms among patients who developed LON than among control subjects.

Previously, we reported that occurrence of LON is associated with a more pronounced and sustained B-lymphocyte depletion and lower levels of serum IgM following rituximab than in matched control subjects with a similar history of previous antirheumatic therapy [3]. In the present study with an extended control group of patients who did not develop LON following rituximab therapy, the presence of lower serum IgM levels in the LON group than in non-LON control subjects was confirmed. In the present study, we further found that the homozygosis for the high-affinity V allele of *FCGR3A* was correlated to lower IgM levels over time compared with the low-affinity F allele.

These findings suggest that the high-affinity V allele of *FCGR3A*, leading to an enhanced binding of rituximab to FcγRs, might confer deeper and more prolonged B-lymphocyte depletion that may result in both LON and more pronounced and prolonged decrease of serum IgM. Whether monitoring of serum IgM levels in patients with a certain genotypic polymorphism may be useful for prediction of LON development is to be addressed in larger prospective studies.

In this study, we found no significant association between LON and serum BAFF levels or *BAFF* promoter polymorphisms (which may influence serum BAFF levels), but the number of available blood samples was limited, and only a few samples were drawn during the LON period. Patients with the *BAFF* −871T/T allotype, however, had a better clinical response with a longer flare-free survival than patients with the *BAFF* −871C/T or C/C allotype. Though caution is needed in interpretation of the findings of our analysis in patients with different rheumatic diseases, our findings are in line with a reported improved response to rituximab in patients with seropositive RA carrying the *BAFF* −871T/T allotype [20]. To avoid findings by chance, we did not proceed with a subanalysis in patients possessing autoantibodies. Further study of BAFF regulation in the context of severe acute neutropenia is of particular interest because BAFF is produced largely by neutrophils and monocytes and because BAFF expression is augmented by G-CSF [37], which is often used for patients who present with infectious complications due to LON.

The present analysis demonstrated that the occurrence of LON and the *FCGR3A* 158V/V genotype both were related to a longer time to flare of the rheumatic diseases. Transient neutropenia is recognized in idiosyncratic drug-induced neutropenia and agranulocytosis [38]. Our findings of lower serum IgM levels in patients who developed LON and lower serum IgM levels in association with the *FCGR3A* V allele, together with a longer time to flare, indicate that the phenomenon of LON is of a different nature than drug-induced agranulocytosis and that LON is likely explained by biological mechanisms of rituximab action modified through *FCGR* genotypes (i.e., a more pronounced B-cell depletion).

Although we report a potentially important finding, LON is a rare clinical phenotype, and there were only 11 cases of LON associated with rituximab in our study. Our results should be verified in larger cohorts performed in disease-specific patient populations with a larger number of cases presenting with LON. Statistical power to evaluate the effect of genetic variation on a rare event such as LON could be improved by performing meta-analyses of combined data from several cohorts. The small number of cases in the present study probably limited the chance of detection of effects of some genotypic polymorphisms (type II error), but the main results appear to be in line with those in previous reports. The strength of the study lies in its advantage of a large cohort of 214 consecutive patients treated with rituximab who were followed prospectively with a variety of important clinical and laboratory data. The possible confounding effects of exposure to other significant drugs was minimized (as could not formally be overcome in the observational study) by individual matching of the LON cases and control cases by the cumulative number of previously used DMARDs; use of cytotoxic and biologic therapies; and concurrent treatment with DMARDs, cyclophosphamide, and corticosteroids. The patients represented a typical, clinic-based, “real-life” patient population treated with rituximab; thus, the selection bias was minimal because our center provides care for patients with the whole spectrum of rheumatic diseases.

## Conclusions

A novel association between the occurrence of LON and the possession of a SNP in the *FCGR3A* gene adds to the understanding of the LON phenomenon in patients with rheumatic disease treated with rituximab. Our results suggest that LON is a result of the intrinsic efficacy of rituximab. The presentation with LON may indicate a longer biological and therapeutic activity of rituximab modulated by certain genotypic polymorphisms. Our findings suggest that the prognosis of therapeutic efficacy and risk for a potentially severe adverse reaction following rituximab therapy may benefit from including genotypic characterization. The findings of the study fuel the need for further comprehensive mechanistic studies on therapeutic activity of rituximab and other antibody agents.

## Abbreviations

AAV: Antineutrophil cytoplasmic antibody-associated vasculitis
ADCC: Antibody-dependent cellular cytotoxicity
BAFF: B-cell-activating factor
DMARD: Disease-modifying antirheumatic drug
ENT: Ear, nose, throat
FcγR: Fcγ receptor
G-CSF: Granulocyte colony-stimulating factor
IgG: Immunoglobulin G
IgM: Immunoglobulin M
LON: Late-onset neutropenia
NK: Natural killer
RA: Rheumatoid arthritis
SLE: Systemic lupus erythematosus
SNP: Single-nucleotide polymorphism
